# The long-term progression of macrodactyly

**DOI:** 10.1016/j.jpra.2021.10.004

**Published:** 2021-10-23

**Authors:** Merel L.E. Stor, Max M. Lokhorst, Sophie E.R. Horbach, Chantal M.A.M. van der Horst

**Affiliations:** Department of Plastic, Reconstructive and Hand Surgery, Amsterdam University Medical Centers, University of Amsterdam, Meibergdreef 9, 1105, AZ, Amsterdam, The Netherlands

**Keywords:** Macrodactyly, macrodystrophia lipomatosa, overgrowth, PIK3CA

## Abstract

**Background:**

Macrodactyly is a rare congenital disorder of overgrowth affecting the digits of the upper or lower extremity. Mostly, patients are surgically treated during childhood to reduce the digit or to stop growth. There are no standardized guidelines for the treatment and follow-up of macrodactyly. Consequently, follow-up may not be regularly scheduled into adulthood.

**Methods:**

A retrospective, descriptive analysis of patients with the long-term progression of macrodactyly who presented at our tertiary referral hospital between July 2018 and March 2020 was performed. All patients from our local macrodactyly database were screened for progression of macrodactyly since adulthood; this resulted in four patients. The aim of these case series is to highlight the clinical features and disease course at long-term follow-up.

**Results:**

All patients were surgically treated during childhood and showed progression of tissue overgrowth during adult life. All patients developed severe secondary degenerative bone changes in macrodactyly affected digits, such as ankyloses of joints, new bone formation, and bony spurs. Subsequently, tissue overgrowth and degenerative bone changes led to functional problems.

**Conclusion:**

Patients with macrodactyly may experience growth during adult life, which may progress to deforming changes. Consequently, patients should be informed about the possible growth, and the progressive growth should be monitored.

## Introduction

Macrodactyly is a rare congenital disorder of overgrowth affecting the digits of the upper or lower extremity.^1^ Digital enlargement may involve all types of mesenchymal tissue, which involve muscle, bone, and predominantly fibro-adipose tissue. Not only fingers and toes but also adjacent parts of the hand or foot may be affected. Macrodactyly can be classified by the rate of growth, either static, growing proportionally with the hand or foot, or progressive, growing faster than the rest of the limb. Thus, macrodactyly encompasses a wide range of clinical phenotypes, with the growth rate, location, and extent of overgrowth varying greatly between patients.

Patients may encounter functional problems and difficulty in walking due to enlargement of the foot in length and width.^2^ Additionally, complaints of cosmetic disfigurement are often experienced.^3^ At a later stage, secondary functional problems may develop such as secondary osteoarthritis and compression of neurovascular structures.^4^

Recently, somatic gain-of-function mutations in the PIK3CA gene were detected in the affected tissue in patients with macrodactyly.^5^, ^6^, ^7^, ^8^ Mutations in the PIK3CA gene were also found in several overgrowth disorders, which are now grouped as PIK3CA-related overgrowth spectrum (PROS) disorders.^9^^,^^10^

Current medical management of macrodactyly is mainly ablative and not based on molecular targets. Treatment consists of debulking a part of the enlarged digit, epiphysiodesis to stop longitudinal skeletal growth, and amputation when the enlarged parts are no longer functional.^11^ However, due to the rarity of the disease and the highly variable manifestations of macrodactyly, there are no standardized guidelines for treatment and follow-up of macrodactyly.^11^ As often a stable situation is achieved during childhood, follow-up may not be regularly scheduled into adulthood. However, to date, little is known about the long-term disease course of macrodactyly and the possible progression of overgrowth. The aim of this study is, therefore, to highlight the clinical features and disease course of macrodactyly at long-term follow-up.

## Methods

Patients with the long-term progression of macrodactyly who presented at our tertiary referral hospital between July 2018 and March 2020 were included. All patients from our local macrodactyly database were screened for progression of macrodactyly since adulthood; this resulted in four patients. Data were extracted from the electronic patient files, which included: patient age, gender, anatomical location of macrodactyly, presence of symptoms, comorbidities, previous treatments, imaging results, histopathology results, and genetic test results. Our institutional review board approved a waiver of consent for this study and written informed consent was obtained from all patients. The guidelines of the Strengthening the Reporting of Observational Studies in Epidemiology (STROBE) Statement were followed.^12^

## Results

All patients are listed in Table 1.

**Table 1:** Patient characteristics

Case (gender, age)	Affected body part	First surgery	Age at first surgery	Additional surgeries	Age at additional surgeries	Reason for consultation	Imaging	Following treatment	Genetic analysis
Case 1 (male, 59)	1st, 2nd, 3rd toe of the left foot	Amputation 1st, 2nd toe through MTP joint	1	Soft tissue debulking	3, 4, 12, 17	Plantar swelling and functional problems	Bony spurs MT II toward plantar side and synostosis with MT I. Ankylosis Lisfranc and partial ankylosis Chopart	Amputation through the Chopart joint (planned)	No
Case 2 (female, 33)	1st, 2nd toe of the left foot	Amputation 2nd toe through MTP joint	7	None	-	Progressive growth of the 1st toe	1st toe with bone overgrowth and bone deformation of the phalanxes and around MTP joint. Ankylosis IP joint	Removal bone exostosis and soft tissue debulking (planned)	No
Case 3 (female, 44)	2nd and 3rd toe of the left foot	Amputation 3rd toe through MTP joint	4	None	44	Progressive growth of the 2nd toe and pain forefoot	Bone deformation MT and proximal phalanx of 2nd toe. Ankylosis MTP joint of the 2nd and 3rd toe	Amputation 2nd toe and removal bone deformation	PIK3CA mutation
Case 4 (female, 47)	Thumb and index finger of the right hand	Soft tissue debulking of the thumb	18	Shortening proximal phalanx, arthrodesis IP and MCP joint	38	Progressive swelling and worsening of function of the thumb and index finger	Juxta-articular new bone formation CMC, MCP, and PIP joint of the thumb and index finger, and around scaphoid, trapezium, and trapezoid	Splint	No
				CTR and correction osteotomy thumb	38				

D = Digit, MTP = Metatarsalphalangeal, MT = Metatarsal, IP = Interphalangeal, CMC = Carpometacarpal, MCP = Metacarpophalangeal, PIP = Proximal interphalangeal.

### Case 1

A 59-year-old male with macrodactyly of the first, second, and third toe of the left foot presented at our hospital. At the age of 1, the first and second toe were amputated through the metatarsophalangeal (MTP) joint. At the age of 3, 4, 12, and 17 years, he underwent additional soft tissue debulking surgery. These procedures took place at another hospital; thus, comprehensive information is lacking.

**Figure 1:**
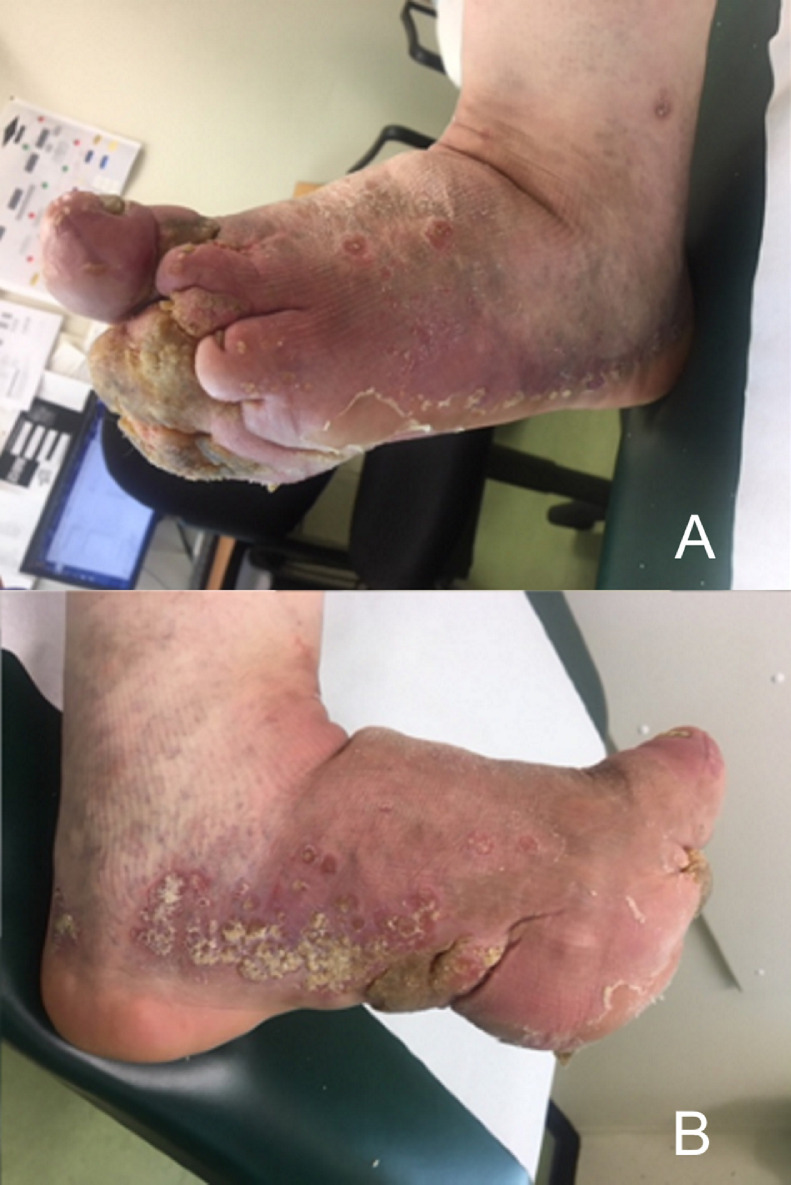
Lateral (A) and medial (B) view of the left foot with macrodactyly of case 1. Note the massive plantar swelling, hyperkeratosis, and psoriatic lesions.

At the age of 54, he noted severe enlargement of the left forefoot. He experienced a total loss of sensory function of the forefoot and therefore did not feel any pain. He encountered functional problems and was unable to walk far distances. Additionally, he suffered from recurrent erysipelas of the foot, once complicated with osteomyelitis. On clinical examination, a deformed left forefoot was seen with a massive swelling at the plantar side and substantial hyperkeratosis. Also, he developed psoriatic lesions on the medial and lateral side of the foot (Figure 1).

**Figure 2:**
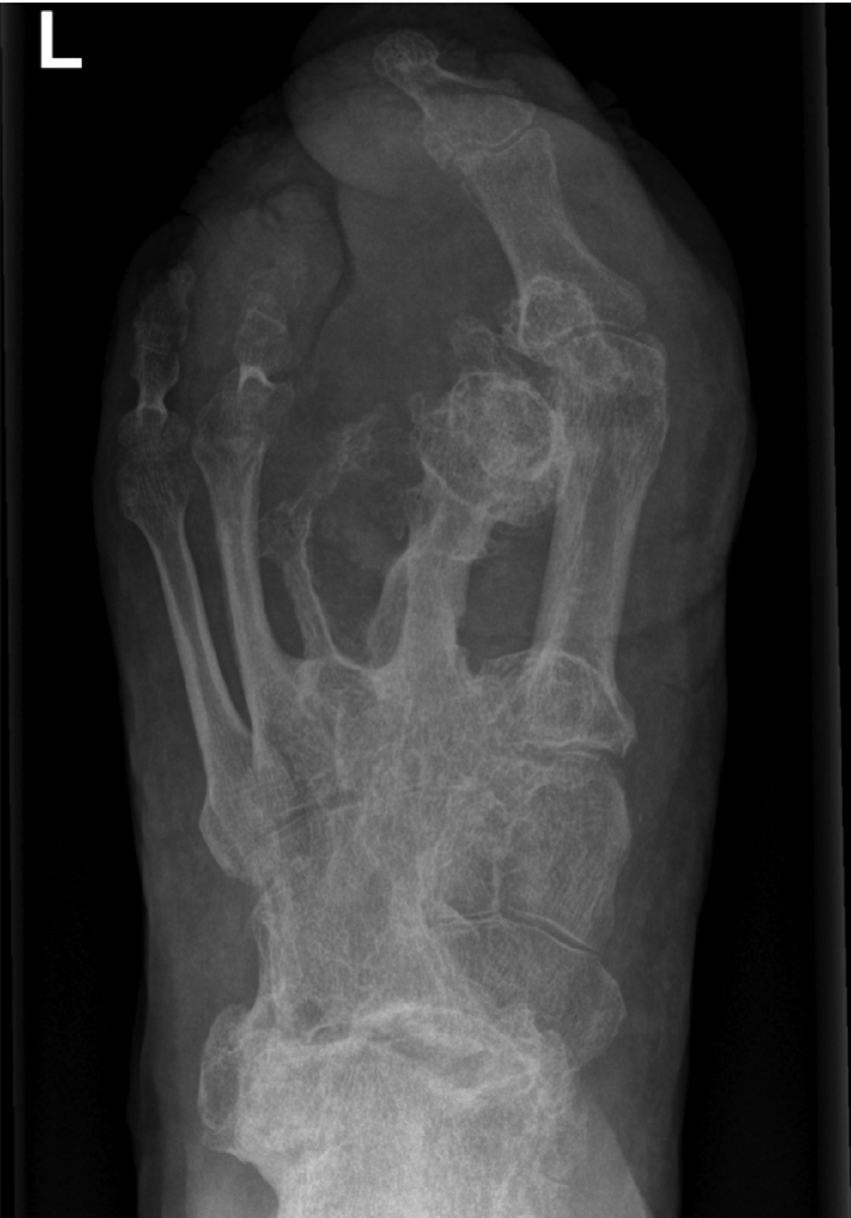
Conventional A-P radiograph of the left foot of case 1 showing a synostosis between metatarsal I and II. Status after amputation of the first and second toe in 1962 and additional soft tissue debulking surgery in 1964, 1965, 1973, and 1978.

**Figure 3:**
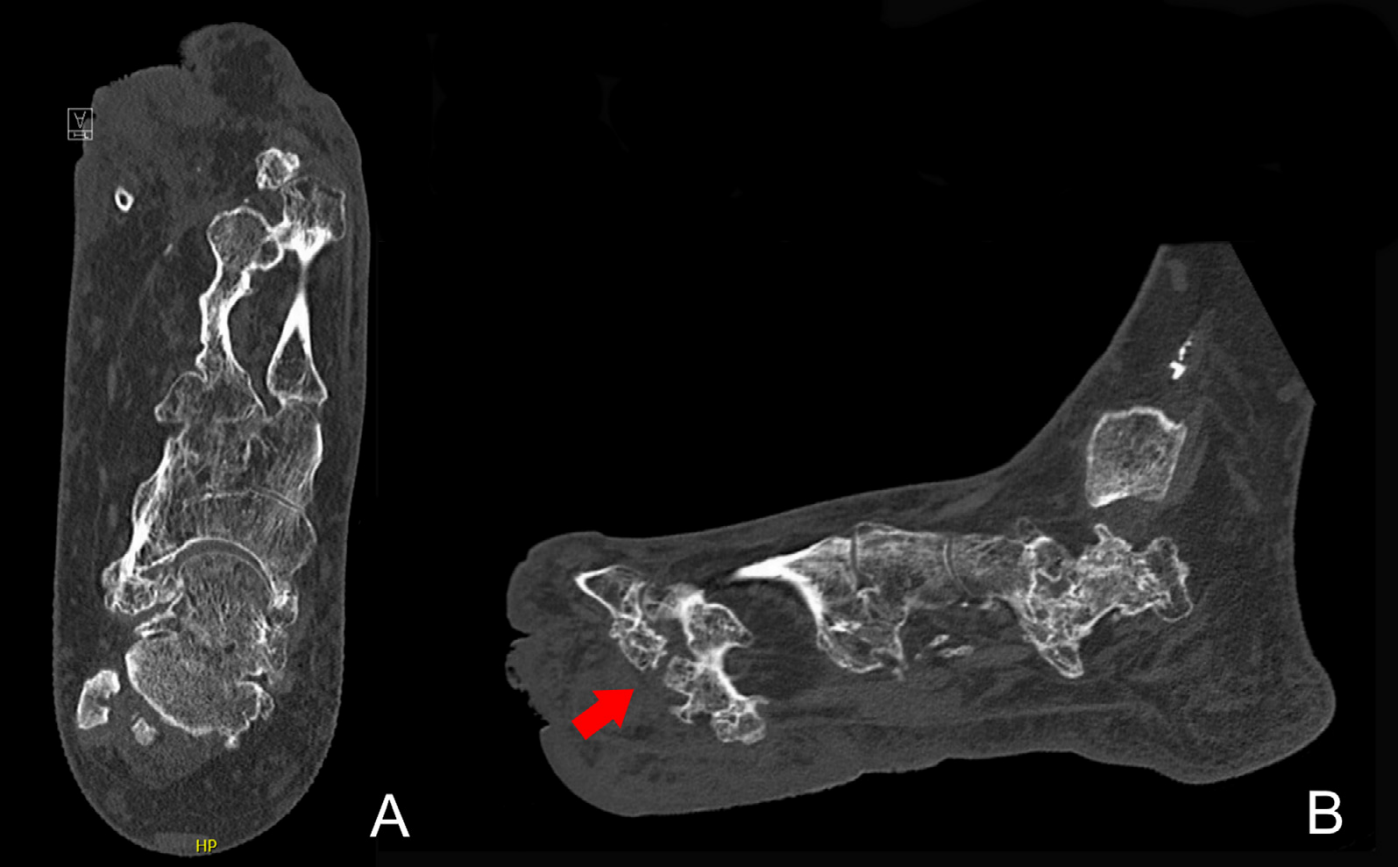
CT-scan of the left foot of case 1. Status after amputation of the first and second toe in 1962 and additional soft tissue debulking surgery in 1964, 1965, 1973, and 1978. 3A: Transversal view showing a synostosis between metatarsal I and II. 3B: Sagittal view with a visible bone spur from metatarsal II toward the plantar side (arrow).

Conventional radiograph and computed tomography (CT) scan of the left foot showed substantial hypertrophy of fat tissue, muscles, and bone tissue in comparison with the right foot. In particular, metatarsal II showed bone overgrowth with bony spurs toward the plantar side and synostosis with metatarsal I. Ankyloses of the Lisfranc joint were visible between the medial, intermediate, and lateral cuneiform bones and metatarsals I, II, and III. Additionally, ankyloses of the Chopart joint were visible, with partial fixation between the talus and navicular bone and partial fixation between the calcaneus and cuboid bone (Figure 2 & 3).

Because of the substantial growth of his left foot and mobility disabilities, he needs additional surgical treatment. Currently, a Chopart amputation is planned because of the best functional advantages.

### Case 2

A 33-year-old female with macrodactyly of the first and second digit of the left foot. At the age of 7, the second toe was amputated through the MTP joint, and the soft tissue of the first toe was debulked.

**Figure 4:**
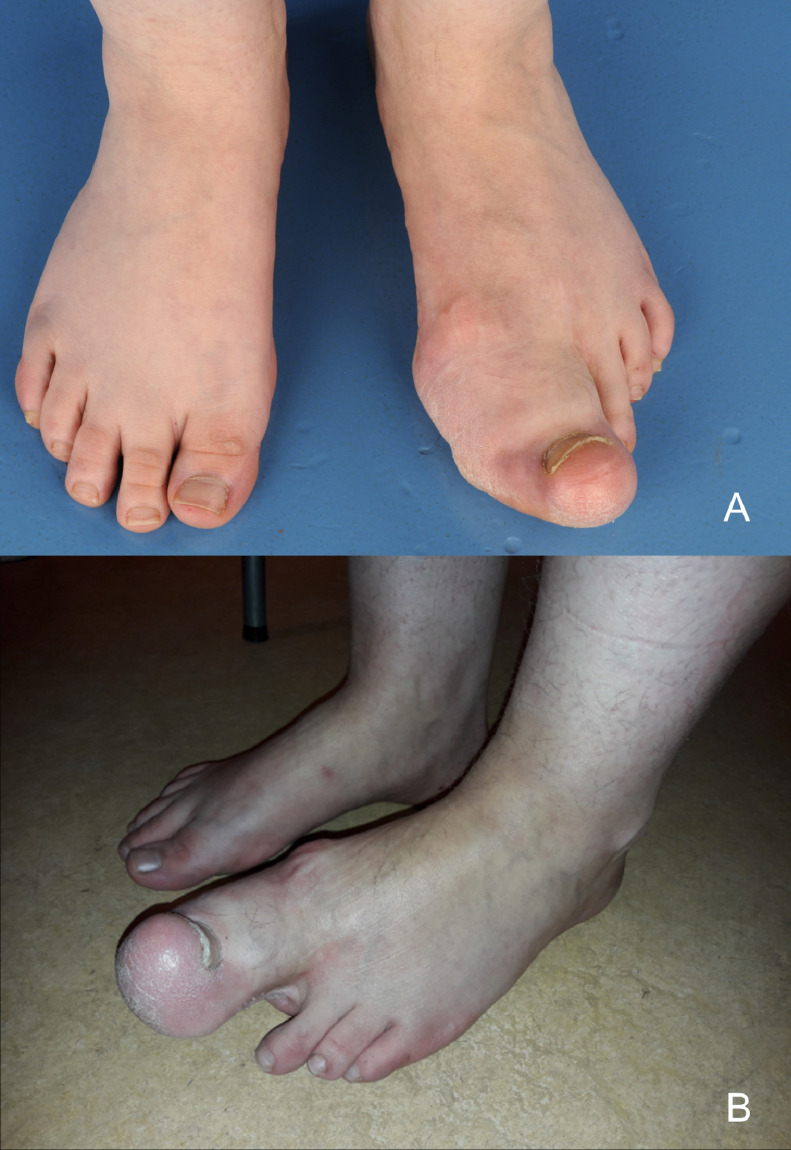
Macrodactyly of the first digit of the left foot of case 2. Progression of overgrowth between age 26 years (4A) and age 31 years (4B).

**Figure 5:**
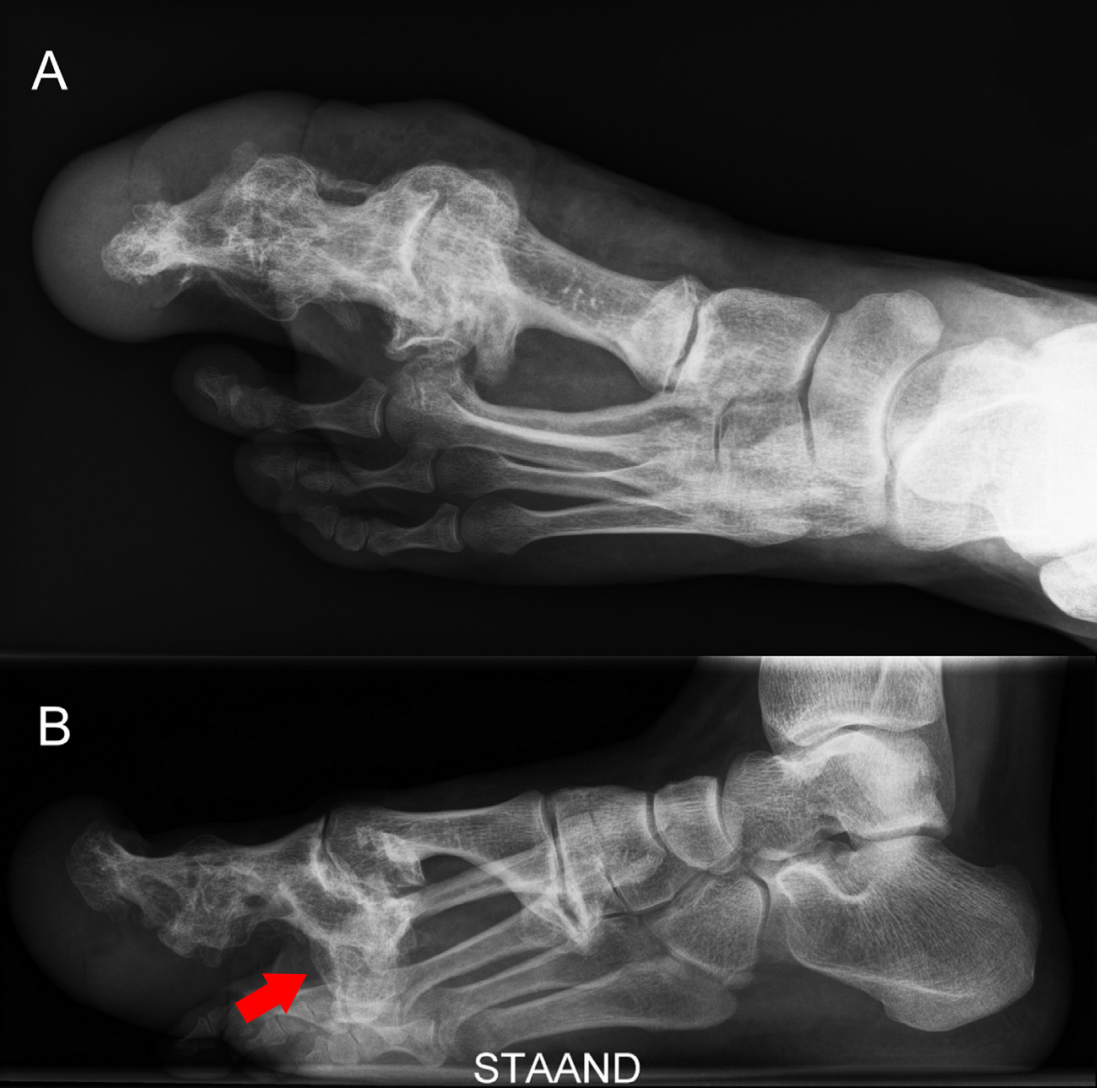
Conventional radiographs of the left foot of case 2. Status after amputation of the second toe through the MTP joint and soft tissue debulking of the first toe in 1993. A: A-P radiograph showing significant bone overgrowth and deformation of the phalanxes and around the MTP joint of the first toe. B: Lateral radiograph in standing positions, notice the substantial bony formation on the plantar side of the foot (arrow) and thereby tilt of the foot.

Since age 30, the first digit grew progressively. She felt slight discomfort lateral of the second digit without experiencing any real pain. On clinical examination, the first digit was enlarged with a substantial swelling of the plantar side (Figure 4). The MTP joint and the interphalangeal (IP) joint were unable to move. Imaging showed an enlarged and deformed first digit with ankyloses of the IP joint and striking soft tissue overgrowth. Further, a severe bone deformation was visible, especially of the phalanxes and around the MTP joint (Figure 5).

Because of the rapid growth of the first digit and the additional functional problems, she will undergo another surgery.

### Case 3

A 44-year-old female with macrodactyly of the second and third toe of the left foot and concomitant syndactyly between these toes. Previously, at the age of 4 years, the third toe was amputated through the MTP joint and simultaneously the syndactyly was resolved.

**Figure 6:**
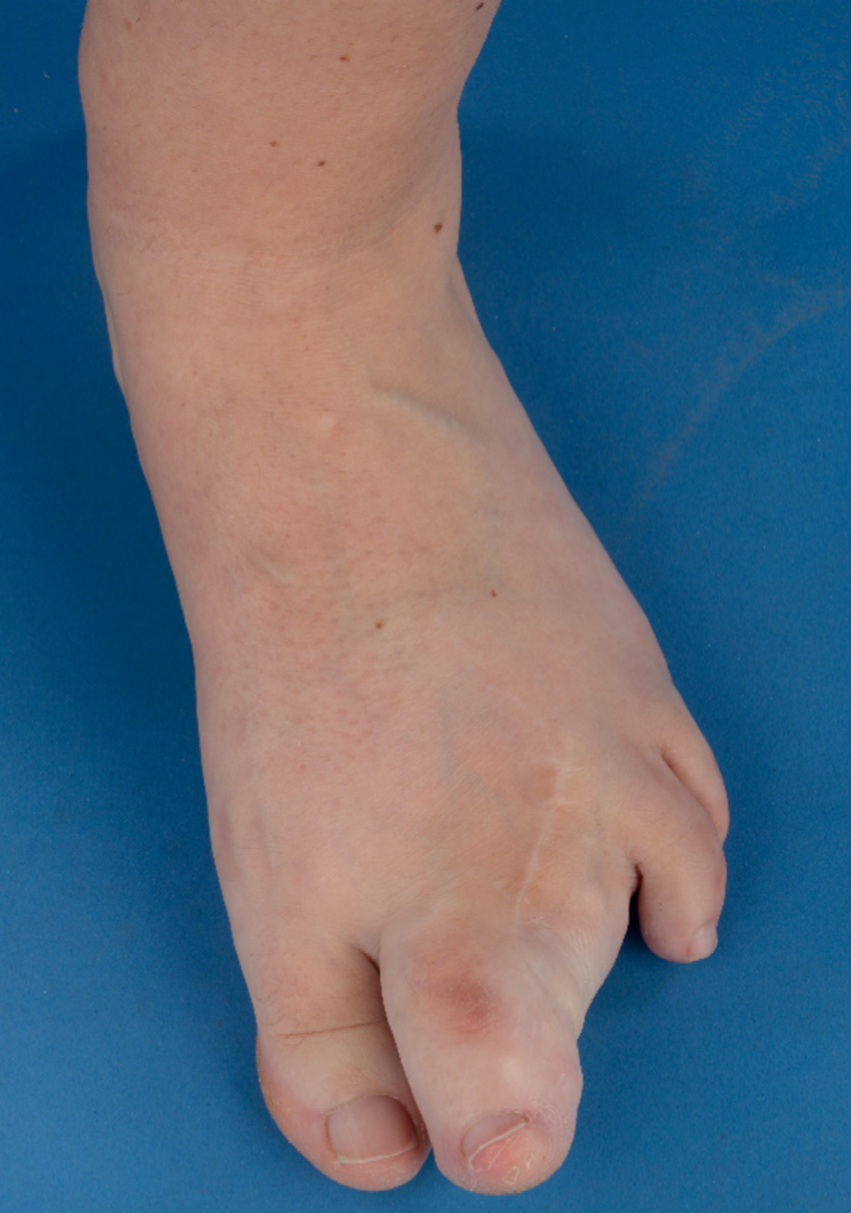
Left foot of case 3 with macrodactyly of the second toe.

**Figure 7:**
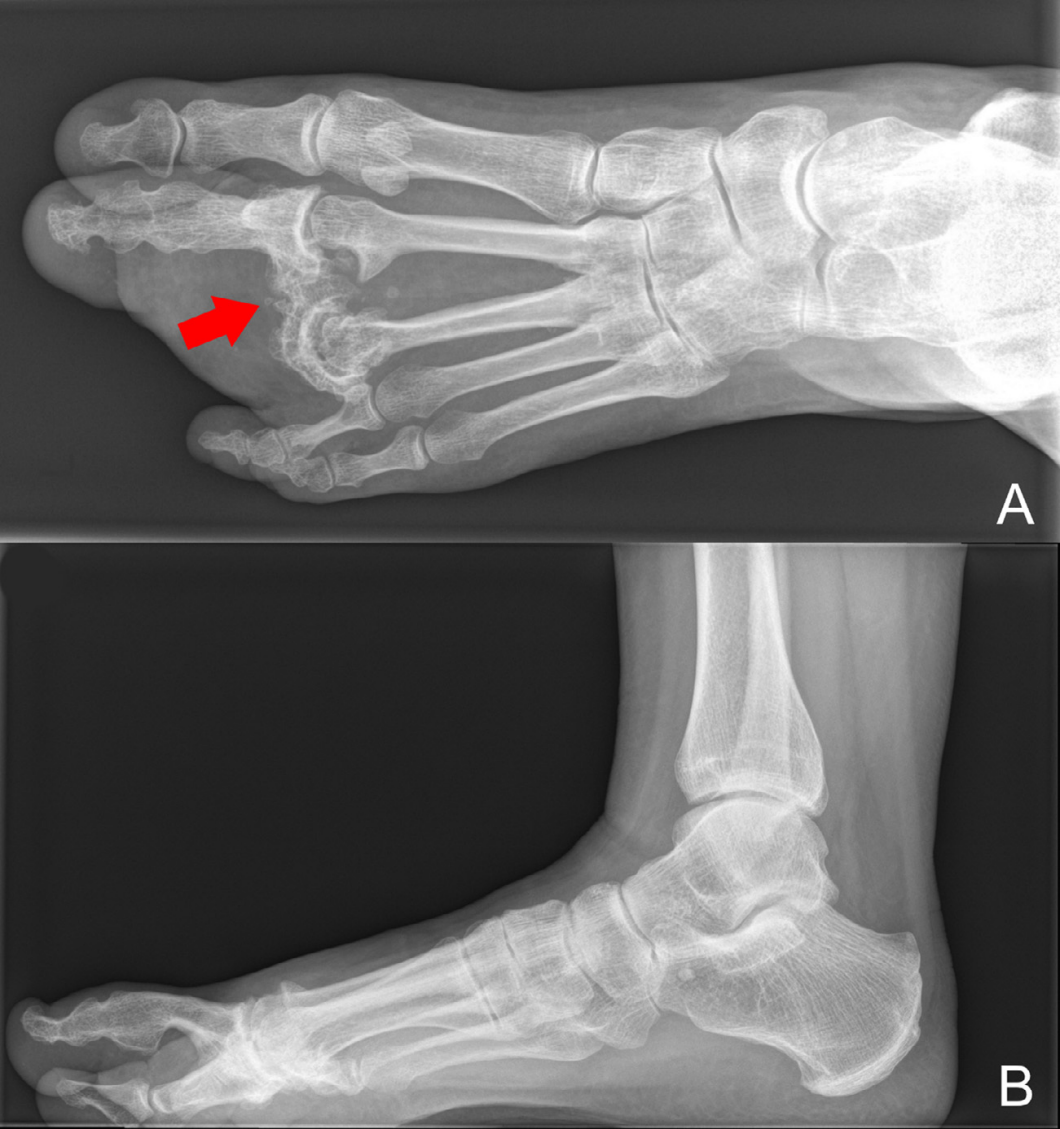
Conventional radiographs of the left foot of case 3. Status after amputation of the third toe through the MTP joint in 1980. 7A: A-P radiograph showing bone exostosis and osteoarthritic deformed bones of the second and third toe (arrow). 7B: Lateral radiograph showing elevation of the second toe because of the plantar swelling.

Several years before she visited our clinic, she experienced increasing pain in her forefoot and was unable to wear her orthopedic shoes because of the fast growth of the foot. The pain occurred dorsal and lateral of the second toe and radiated to the fourth and fifth toe. On clinical examination, her second toe was enlarged with a plantar swelling (Figure 6), and her left foot was three cm wider in comparison with her right foot. Her gait was impaired by the enlargement of her second toe, which was not able to touch the ground. Conventional radiographs of the left foot showed severe osteoarthritis and ankyloses of the MTP joint of the second and third toe. Additionally, exostosis of the metatarsal II toward the third toe was visible (Figure 7).

Considering the progressive growth of the second toe and accompanying complaints, she underwent further surgery on her left foot. The second toe was amputated distally from the MTP joint and osteoarthritic deformed bone was removed. The second toe was sent in for genetic analysis, which showed a somatic mosaic mutation in PIK3CA. The pathogenic variant c.3140A>T p.(His1047Leu) was detected with a variant allele frequency (VAF) of 25%. The surgical intervention led to pain reduction and increased functioning of her left foot.

### Case 4

A 47-year-old female, known with macrodactyly of the thumb and index finger of the right hand. At the age of 18 years, the first surgery took place with soft tissue debulking of the thumb. However, post-operatively she experienced more functional problems. At the age of 38, she underwent further surgery of the thumb including soft tissue debulking, shortening of the proximal phalanx, and arthrodesis of the IP joint and metacarpophalangeal (MCP) joint. In the same year, a carpal tunnel release and a correction osteotomy of the thumb were performed, which led to an improved position of the IP joint arthrodesis. Nevertheless, she noticed an increase in swelling and tingling of the thumb and index finger.

At age 47, she presented at our hospital because of the progression of swelling and worsened functioning of the thumb and index finger. For a few months, her thumb, index finger, and wrist were painful. On examination, a swelling was seen of the volar, radial side of the hand. Solid nodules were present at the MCP joint and proximal interphalangeal joint (PIP) of the index finger. Her wrist showed a position of 20 degrees ulnar deviation, and flexion and extension were limited.

**Figure 8:**
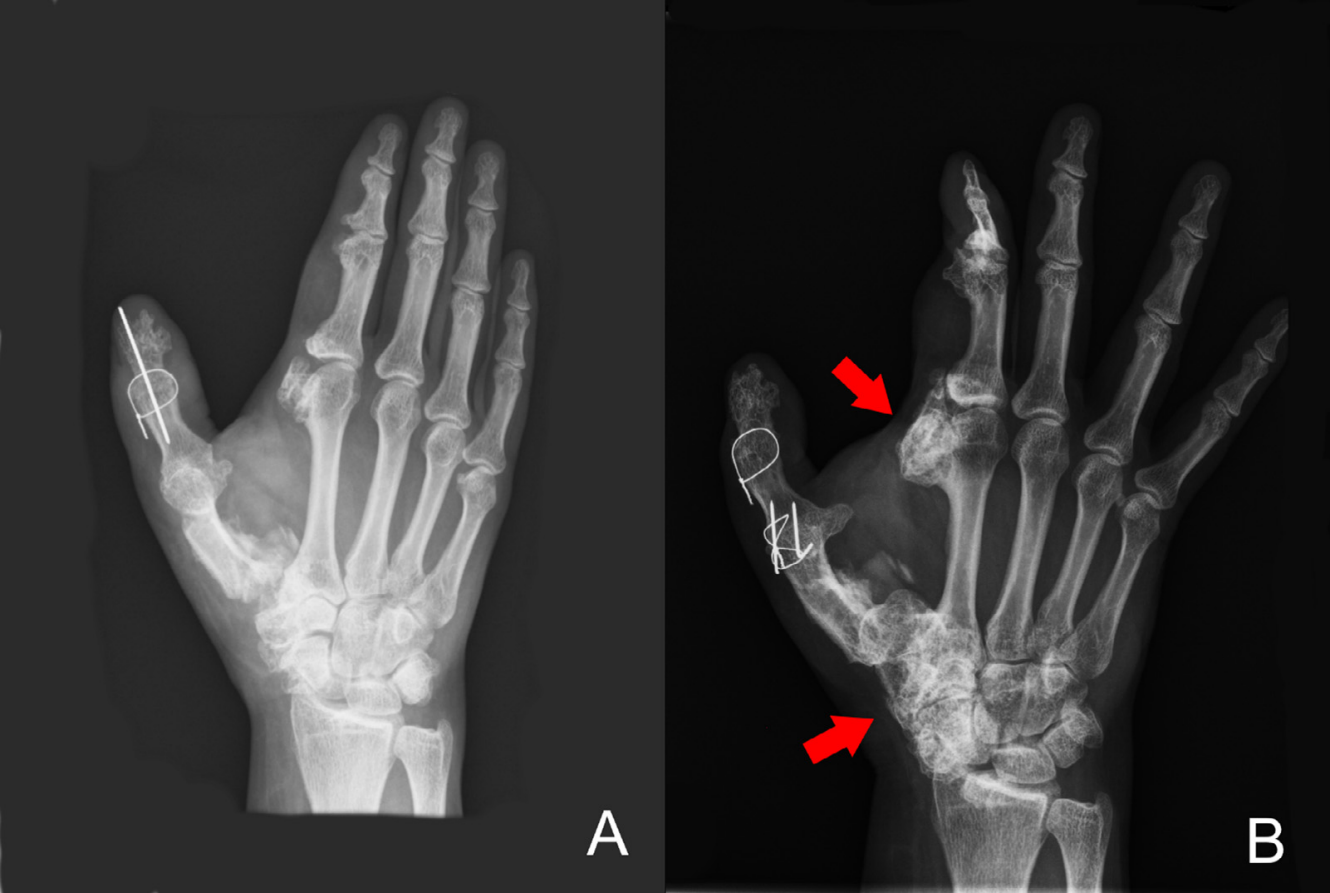
Conventional radiograph of the right hand of case 4. Status after soft tissue debulking surgery of the right thumb in 1989, additional soft tissue debulking, shortening of the proximal phalanx, and arthrodesis of the IP joint and MCP joint of the thumb in 2009, and correction osteotomy of the thumb in 2009. The current radiographs are showing progression of juxta-articular new bone formation between age 38 (8A) and age 47 (8B). In particular, the thumb and index finger (upper arrow) and the scaphoid, trapezium, and trapezoid (bottom arrow) show severe progression.

Conventional radiographs of her right hand showed complete consolidation of the IP and MCP joint arthrodesis of the thumb. Remarkable juxta-articular new bone formation was present in the thumb and index finger of the carpometacarpal (CMC) joint, MCP joint, and PIP joint (index finger). Also, new bone formation was visible of the scaphoid, trapezium, and trapezoid (Figure 8).

Given her functional problems, she was referred to a rehabilitation specialist. Subsequently, a splint was made for her right hand to improve her abilities. Additionally, she was referred to a clinical geneticist. However, she was lost in follow-up and genetic analysis of the affected tissue did not take place.

## Discussion

Four macrodactyly cases with the severe long-term progression of overgrowth were described. All patients experienced rapid growth of the affected digit long after treatment. The results of this study show that tissue overgrowth can progress excessively during adult life. The long-term follow-up of these cases of macrodactyly provides useful insights into the condition.

A remarkable finding was the degenerative and deforming bone changes. The phalanges and metacarpals in our patients were expanded and deformed at their distal ends. This may be explained by the periosteum being studded with nodules consisting of chondroblasts and osteoblasts, which are more numerous toward the end of the phalanges and account for the distal osseous enlargement.^13^ Furthermore, our cases demonstrated new bone formation and the development of bony spurs. A possible explanation for these bony changes could be a misalignment of articular surfaces, which results in severe secondary degenerative joint changes with new bone formation.^14^

In particular, patient 1 developed exceptional bony changes; however, other factors may have contributed to the deformation of the foot. Chronic osteomyelitis may lead to reactive new bone formation, bone deformation, and ankylosis.^15^ Other longstanding cases, described in the literature, showed comparable severe bone changes and newly formed bone synostosis between multiple digits.^16^^,^^17^ It is unclear if surgical treatment during childhood also contributes to the expanded growth and degenerative bone changes. All of our cases were surgically treated during childhood and showed severe overgrowth during adult life. However, patient 4 showed also osteoarthritic changes and juxta-articular new bone formation of unoperated areas, such as the index finger and carpal bones.

Not all patients with macrodactyly develop secondary degenerative joint changes. Ishida et al. investigated long-term term results of surgical treatment for macrodactyly of the hand.^18^ They reported that 2 out of 23 patients developed early degenerative changes in the affected joints, after a mean follow-up of 23 years.

Unilateral involvement in macrodactyly is the most common, which was also noted in our patients. Hands and feet are affected with almost equal frequency. In both hands and feet, third digit involvement is the most prevalent, followed by second digit enlargement.^8^ In macrodactyly patients, syndactyly, polydactyly, and clinodactyly may simultaneously be present,^8^ as was also seen in patient 3 with syndactyly.

For a long time, the etiopathogenesis of macrodactyly was poorly understood. However, now it is clear that postzygotic somatic mutations in the PIK3CA/AKT/mTOR pathway may be a cause of macrodactyly.^5^ This pathway is involved in cell signaling, cell growth, differentiation, and proliferation. PIK3CA encodes the p110α catalytic subunit of phosphoinositide 3-kinase (PI3K), which activates AKT and mTOR signaling to promote tissue growth.^19^

However, between patients, growth trajectories vary greatly for unknown reasons. While some patients exhibit excess growth limited to childhood, others have progressive tissue growth during adult life. As the somatic mutation in PIK3CA remains present in the affected tissue, this may promote tissue growth continuously. Eventually, continuous tissue growth may result in the advanced stages of overgrowth. These cases are an example of continuous growth during adult life and illustrate the deforming changes the overgrowth can entail.

After the discovery of somatic mutations in isolated macrodactyly^6^, PIK3CA mutations were observed in multiple patients with macrodactyly. Wu et al. identified a PIK3CA mutation in nine of twelve patients, indicating that a high proportion of isolated macrodactyly patients carry a pathogenic PIK3CA mutation.^7^ However, a negative result of PIK3CA mutations does not necessarily exclude the presence of a PIK3CA mutation. The patient may have another mutation beyond the targeted genomic regions or the mutation may be below the detection rate. In macrodactyly, the highest mutation detection rate is found in adipose tissue, followed by nerve and skin tissue.^7^

PIK3CA somatic mutations have been found in various overgrowth disorders, such as megalencephaly-capillary malformation syndrome, muscular hemihypertrophy, Klippel-Trenaunay syndrome, and CLOVES syndrome.^20^, ^21^, ^22^ The presenting phenotype of the PROS disorders seems to depend on the timing of the somatic mutation, the tissue localization of the mutations, and the location of the mutation in the embryo. Mutations that occur early during embryogenesis will generate many affected daughter cells, potentially of distinct differentiation routes (stroma, fat, smooth muscle, endothelium, etc.), which may result in larger and multiple body segments that are affected, such as in CLOVES syndrome.^10^ A mutation later in embryogenesis will produce lower numbers of mutated cells and yield smaller lesions, such as in macrodactyly.

There exist several limitations in these case reports. These cases are not representative of all longstanding macrodactyly cases. However, this is inherent to the study design, since primarily patients who experience complaints or growth of macrodactyly would revisit the outpatient clinic. Secondly, the natural course of the disease could not be assessed, as the patients visited the outpatient clinic a couple of years after they experienced growth. Yet, this is unavoidable since patients are not monitored regularly during adult life. Unfortunately, we did not have genetic information on all patients. Two patients will undergo surgery soon, where the tissue will be taken for genetic analysis.

In conclusion, our study shows that tissue overgrowth can continue and progress excessively in patients with macrodactyly without monitoring during adult life. Somatic mutations in PIK3CA were identified in patients with macrodactyly and may be responsible for this continuous growth. Clinicians should be aware of the possibility of continuous tissue growth and the deforming changes the progressive overgrowth can entail. Monitoring during adulthood may lead to earlier intervention, which may prevent excessive overgrowth and may preserve function. Therefore, clinicians should inform patients that growth may occur in a later stadium, and patients should receive instructions to revisit the outpatient clinic if worsening of function or growth occurs. Currently, no follow-up guidelines exist. Monitoring patients with macrodactyly regularly every three years is recommended. Follow-up should consist of an evaluation of the function, size, and degenerative changes of the affected digits by physical examination and conventional radiography.
